# Locating the Route of Entry and Binding Sites of Benzocaine and Phenytoin in a Bacterial Voltage Gated Sodium Channel

**DOI:** 10.1371/journal.pcbi.1003688

**Published:** 2014-07-03

**Authors:** Lewis J. Martin, Ben Corry

**Affiliations:** Research School of Biology, Australian National University, Canberra, Australia; Max Planck Institute for Biophysical Chemistry, Germany

## Abstract

Sodium channel blockers are used to control electrical excitability in cells as a treatment for epileptic seizures and cardiac arrhythmia, and to provide short term control of pain. Development of the next generation of drugs that can selectively target one of the nine types of voltage-gated sodium channel expressed in the body requires a much better understanding of how current channel blockers work. Here we make use of the recently determined crystal structure of the bacterial voltage gated sodium channel NavAb in molecular dynamics simulations to elucidate the position at which the sodium channel blocking drugs benzocaine and phenytoin bind to the protein as well as to understand how these drugs find their way into resting channels. We show that both drugs have two likely binding sites in the pore characterised by nonspecific, hydrophobic interactions: one just above the activation gate, and one at the entrance to the the lateral lipid filled fenestrations. Three independent methods find the same sites and all suggest that binding to the activation gate is slightly more favourable than at the fenestration. Both drugs are found to be able to pass through the fenestrations into the lipid with only small energy barriers, suggesting that this can represent the long posited hydrophobic entrance route for neutral drugs. Our simulations highlight the importance of a number of residues in directing drugs into and through the fenestration, and in forming the drug binding sites.

## Author Summary

The treatment of cardiac arrhythmia, epilepsy and pain usually involves blocking the protein channels responsible for initiating electrical activity in nerves and muscles. Current drugs block all such channels, but improved medication requires compounds that can differentiate between the channels present in different parts of the body. Achieving this goal calls for a better understanding of the interactions of current drugs with the proteins. Here we use computer simulation to understand where a local anesthetic and an anti-epileptic bind to a bacterial sodium channel and how they find their way to this position, helping to uncover ways to selectively target different human channels.

## Introduction

Voltage-gated sodium channels (Navs) are transmembrane proteins that initiate action potentials in excitable cells by responding to small depolarizing signals to allow the rapid influx of 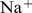 into the cell [1]–[3]. Mutations and aberrant expression of sodium channels are responsible for a range of diseases related to nerve and muscular function including neuropathic pain, cardiac arrhythmia and epilepsy [4], [5]. These conditions often benefit from treatment with drugs that block the passage of 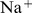 and thereby rein in electrical activity. In clinical settings, the local anesthetic activity of sodium channel-blockers is also used to ease surgery or acute pain resulting from trauma. While there are many molecules capable of blocking sodium channels, most medically relevant drugs are small organic molecules which bind inside the pore to impede ion flow by either directly occluding the ion conduction pathway or by stabilizing a non-conductive channel conformation [3]. The result is reduced signal propagation, which manifests symptomatically as a reduction in seizure occurrence, pain sensation or cardiac excitability.

Sodium channel inhibitors exhibit a wide range of chemical moieties, but common to most of them is the presence of a phenyl ring connected to a basic nitrogen, with some combination of aromatic and aliphatic decorations [6], [7]. Neutral local anesthetics are known to be able to block and leave resting sodium channels when applied from either side of the membrane, a process known as ‘tonic block’. In contrast highly polar or charged compounds can only block the pore after channel opening yielding ‘use dependent’ block that is the basis of anti-epileptic or anti-arrhythmic activity [8], [9]. The observation of multiple modes of drug block led to the suggestion that there are two access routes for drugs to enter the channel: through the activation gate from the cytosol or directly from the lipid bilayer through a ‘hydrophobic route’ [8].

Humans have nine different types of voltage-gated sodium channels, which are preferentially expressed in different tissue [2]. Most sodium channel blocking drugs target all subtypes, but the development of selective channel blockers would allow a new range of clinical applications and a decrease in side effects [7]. For example, there is significant interest in increasing the specificity of drugs for the channel subtypes found in pain receptor neurons as a way to combat chronic pain [10], [11]. However, developing subtype-selective Nav channel blockers requires the generation of in-depth knowledge of the location of binding and the route of entry of existing drugs in order to assess how small sequence differences in the subtypes can be exploited. The presence of a common chemical moiety in sodium channel inhibitors also raises the question of how drugs with similar structure and binding sites, such as local anesthetics and anticonvulsants, have differing therapeutic effects [12]. A better understanding of the specific interactions of these compounds with residues in the channel and their access route may suggest strategies for altering drug kinetics and affinities.

While there are no atomic resolution structures of eukaryotic voltage gated sodium channels (eNavs), the recent publication of a number of structures of homologous channels from bacteria (bNavs) [13]–[17] provides an avenue to understanding how sodium channel blockers work at the molecular level. The first crystal structure of a bNav from *Arcobactor Butzleri*, called NavAb, shows the protein is a tetramer of four identical subunits, with the pore axis running down the central axis [13]. Each subunit contains a pore forming domain consisting of two transmembrane helices (S5 and S6) joined by a linker that forms the narrow selectivity filter at the extracellular end of the pore. Beneath this is a central, water-filled cavity, walled by the S6 helices, where most channel-blocking drugs are known to bind in eNavs [18]–[22]. The pore tapers at the intracellular end to form the activation gate which is closed in this structure so that drugs cannot pass from the cytosol into the central cavity. Recent electrophysiological studies have shown that the local anesthetics lidocaine, ranozaline and benzocaine can block sodium flux in NaChBac, the most well characterised bNav, with comparable affinity to tonic block in eukaryotic channels [9], [23]. Supporting the similarity between the bacterial and eukaryotic channels, uncharged drugs such as benzocaine can block NaChBac when applied extracellularly indicating that the hydrophobic route for drug entry is still present [9]. The published bNav structures present a candidate for this drug access route as they show lateral fenestrations extending to the membrane from the center of the pore, which are large enough to fit small molecules [24], [25], and through which the general anesthetic isoflurane has been seen to pass in simulation studies of a NachBac homology model [26].

Despite the similarities between bacterial and eukaryotic voltage gated sodium channels that may make bNavs good models for studying tonic block, there are also functional and structural differences. NaChBac exhibits far slower gating kinetics than eNavs [27], precluding the study of use-dependent block in which recovery from inactivation must occur faster than drug dissociation. Structurally, bNavs are composed of four identical subunits, whereas eNavs are a single, heterotetrameric protein chain [27], [13]. Although there is common hydrophobic character in the pore, sequence alignments show that residues forming the putative drug receptor site on the internal S6 helix are not conserved between bacterial and eukaryotic Navs [28], and specifically NavAb lacks aromatic residues near the drug binding site that are suspected to be particularly important in use-dependent binding [21].

In order to better understand the mode of action of sodium channel inhibitors, here we use molecular dynamics simulations to examine the route of entry and mechanism of binding in NavAb of two channel-blocking drugs: the local anesthetic benzocaine, and the anti-epileptic phenytoin. Both are small, neutral drugs that bind inside the pore of eNavs [29], [20] and whose blocking effect tapers off after some time [30], [31], indicating they may leave the channel via the observed hydrophobic fenestrations. Benzocaine has been shown to illicit tonic block of NachBac [9]. Equilibrium and biased simulations are used to identify the potential binding sites of these two drugs in the pore, and to quantify the affinity of binding. Accompanying this is a demonstration of drug passage through the hydrophobic fenestration, showing that this pathway is a feasible route of access or escape for neutral channel-blocking drugs.

## Results

### Locating potential drug binding sites

As described above, mutation studies strongly support the notion that local anesthetics bind to residues in the central cavity. For this reason we limit our search for possible binding sites to the channel cavity and fenestrations in order to more extensively sample potential binding pockets (meaning that we will not pick up any sites on the exterior of the protein.) To gain a first appreciation of where the drugs might interact with the protein and to observe drug behaviour inside NavAb, we start our investigation by placing phenytoin or benzocaine in the middle of the central cavity of both the closed and inactivated conformations and allow them to explore the interior of the pore without external influence. Although the size of the cavity is small and the drugs rapidly move about in this region, the timescale of our simulations is shorter than the typical time taken for drugs to block the channel, so it is possible that the drugs will not find their way to the most likely site in the pore. However, this approach does enable us to make a first identification of potential binding pockets which can be verified with the more exhaustive metadynamics search described below. As will be described later, the same binding positions found in these equilibrium simulations are found in two additional independent methods, lending support to them representing likely binding positions in the bacterial channel NavAb.

**Figure 1:**
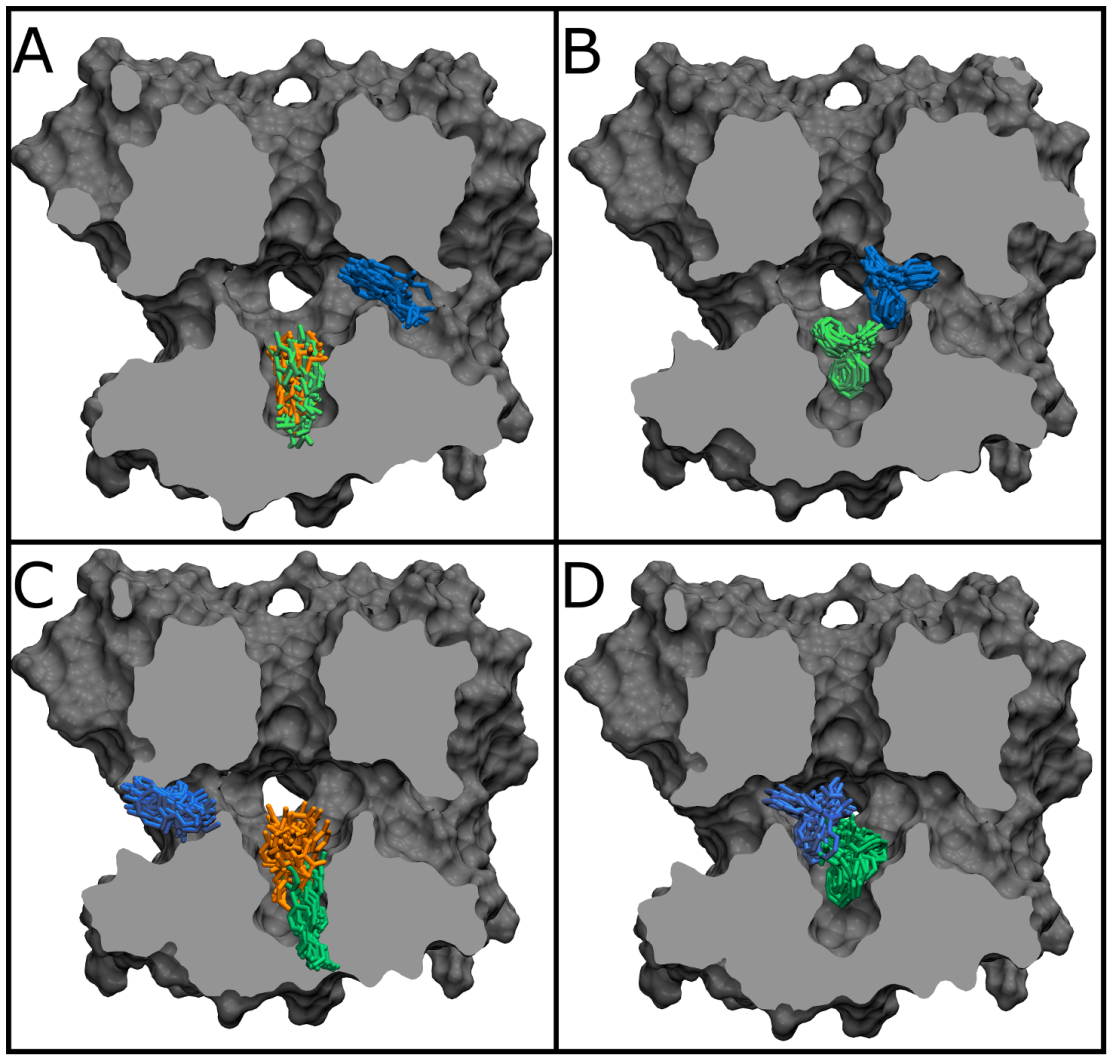
Drug positions in unbiased simulations. 20 Equally spaced snapshots from three unbiased simulations of benzocaine (A and C) and phenytoin (B and D) in the closed (A and B) and inactivated (C and D) NavAb. Each colour represents snapshots from a different simulation, while a single protein conformation is shown in each case.

Fig. 1 illustrates the results of a cluster analysis performed on 3 independent 125 ns simulations starting from different initial drug orientations for each protein/drug combination. The snapshots shown highlight the most commonly occupied locations of the drug in the pore. Both phenytoin and benzocaine explored widely throughout the cavity in the repeated simulations, sampling the majority of one of the four homologous cavity walls, and both drugs experienced stable and enduring association to two sites in the channel cavity. In each of our simulations the drug spends the majority of the time in one of the positions highlighted in Fig. 1, indicating that the timescale to move between stable positions is relatively long and that a single simulation will have difficulty to sample all possible positions. However, we do see a number of events in which drugs move between sites or reorient within a site suggesting that amongst our 12 simulations we are likely to be finding the most stable positions in the pore.

One of the most commonly occupied sites is formed by the hydrophobic pocket above the intracellular activation gate, created by the confluence of the four S6 segments at the pore axis (location shown in green in Fig. 1). The second most common site was in one of the four hydrophobic fenestrations that exist between the protein subunits (blue in Fig. 1). Phenytoin, the bulkier molecule, sampled less deeply into the hydrophobic pockets. In contrast benzocaine extends further into the pockets, and in one case even traveled through the fenestration to bind on the outside of the protein. This event, in which the benzocaine moved outward from the position shown in blue in Fig. 1C (green), is notable in that it demonstrates the possibility of leakage of local anesthetics from an otherwise blocked channel.

### Confirming the binding locations with metadynamics

**Figure 2:**
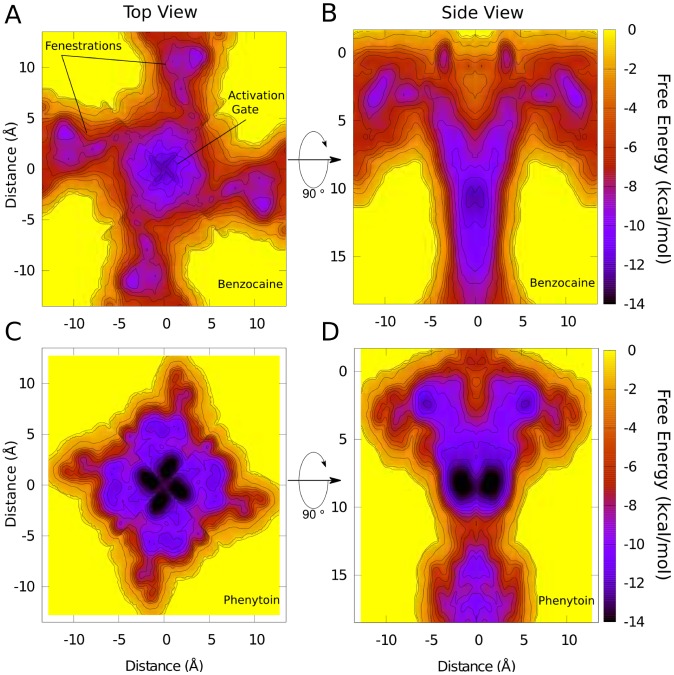
Free energy surfaces for (A,C) benzocaine and (B,D) phenytoin in the NavAb central cavity viewed along the pore axis (A,C) and from the membrane (B,D) obtained from metadynamics simulations. Contours are shown at 1/mol intervals.

Although similar binding positions are seen in each of the 12 independent equilibrium simulations, it is possible that the drugs do not sample all potential sites in the pore. To ensure that we have not missed any important binding site in the equilibrium simulations, metadynamics simulations were run to force the drugs to move throughout the entirety of the pore, providing a much more exhaustive search of the cavity and fenestrations. By adding a wall to prevent the drug moving beyond the external end of the fenestrations selectivity filter or activation gate, the pore forms an enclosed space meaning that the method can also be used to obtain the relative free energy of each position in the pore. The free energy surface for benzocaine (Fig. 2) shows two minima, which correspond to the binding sites observed in the unbiased simulations. Binding at the activation gate is found to be slightly more favourable (by <3 kcal/mol) than at the entrance of the fenestration. This surface also indicates the relative ease with which benzocaine can pass through the fenestration, with barriers of only a couple of kcal/mol to move out from the fenestration binding site to the outside of the protein. Phenytoin shows one clear minimum in the activation gate with a second small minimum present near the hydrophobic fenestration. Remarkably, both drugs experience relatively low barriers to push through the closed activation gate (see barriers at the bottom of Fig. 2. B&D), although the absence of the C-terminal domain in the simulations may be aiding this. It can be seen from the range of of x, y and z coordinates represented in the free energy surfaces (Fig. 2) that the drug moves through all possible positions in the cavity and fenestrations. In Fig. S1 we plot the drug orientation as a function of time, which shows that both drugs sample all possible orientations. The exhaustive search undertaken with the metadynamics simulations confirms the binding poses found in the equilibrium simulations and indicate that for both drugs, binding in the NavAb activation gate is stronger than in the fenestrations.

### Characterising the drug binding sites

**Figure 3:**
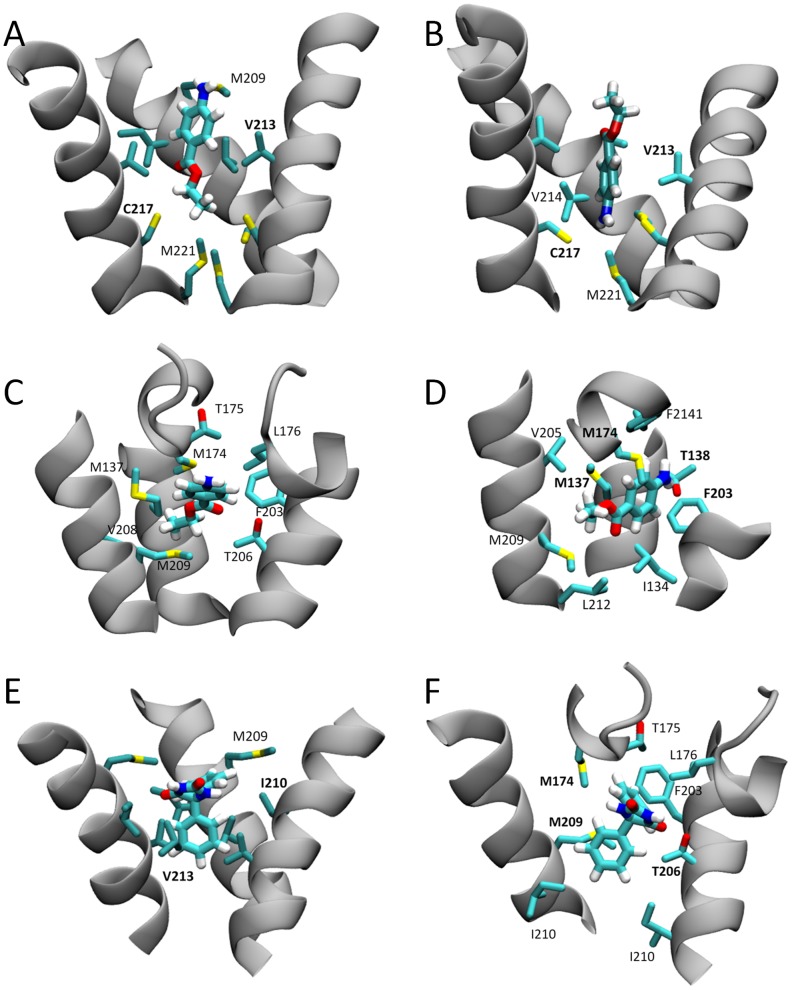
Snapshots of the most commonly sampled binding poses. The drug and surrounding residues are shown. The residues with the strongest interactions with the drug are named in bold. (A) benzocaine in the activation gate with amine pointing at the central cavity. (B) benzocaine in the activation gate with amine pointing down. (C) benzocaine in a fenestration with amine pointing to the central cavity. (D) Benzocaine in a fenestration with amine pointing toward the lipid. (E) Phenytoin in the activation gate. (F) Phenytoin in a fenestration.

**Figure 4:**
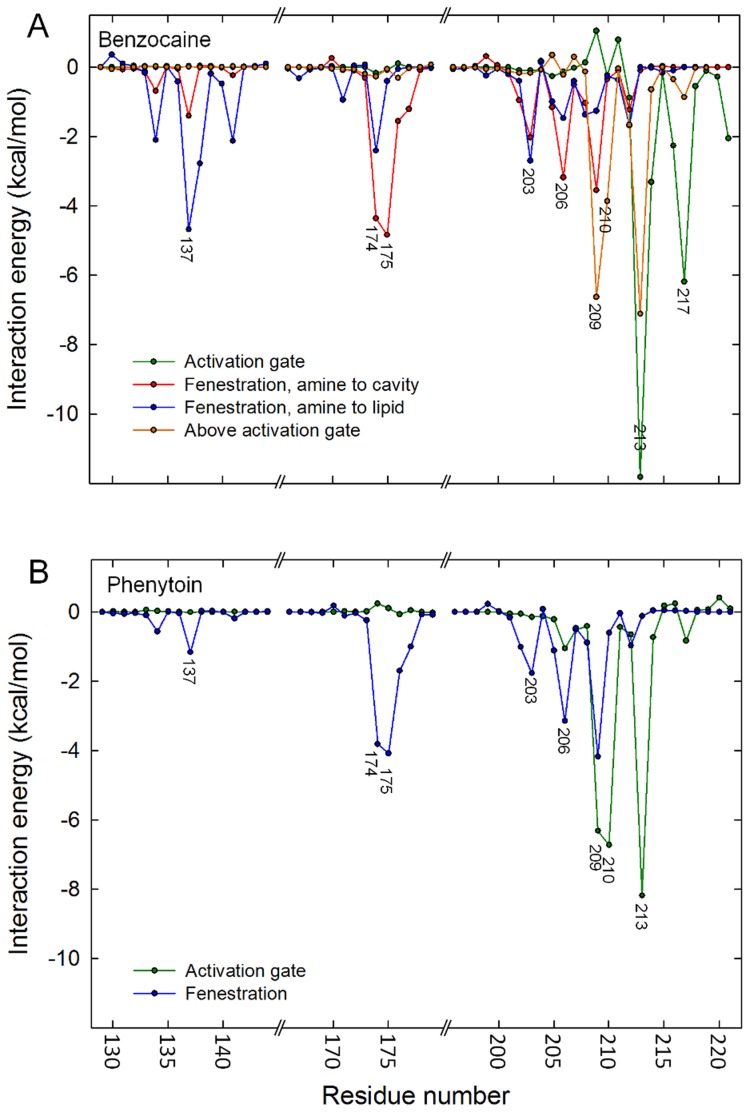
Drug-protein interactions in each site. The interaction energies for (A) benzocaine and (B) phenytoin with residues lining the channel lumen when the drug is in one of the commonly occupied clusters. Four significantly different cluster are shown for benzocaine, corresponding to those pictured in Fig. 1 & 3. Two clusters are shown for phenytoin corresponding to binding at the activation gate (green) and fenestration (blue). Residues from regions not having significant interactions with the drugs are omitted.

Representative snapshots of each of the identified binding sites (taken from the cluster analysis) are shown in Fig. 3 and Fig. S2. In each potential binding site, non-polar moieties of the drug extend into the hydrophobic pockets of the channel cavity. While it is most common to find the polar amine of each drug solvated by water in the central channel cavity, benzocaine can sit in either orientation in the activation gate or fenestration as shown in Fig. 3 A–C. Phenytoin, on the other hand, always buries one of the phenyl rings deep into a hydrophobic pocket. To further characterise the sites, the interaction energy was determined between each drug and each protein residue (Fig. 4). As can be seen in both Figs. 3 and 4, binding in the activation gate is primarily composed of interaction with residues M209, I210, V213 and I217. Binding in the hydrophobic fenestration is primarily composed of interactions with S6 residues T206 and M209 and P2 residues M174, T175 and L176, as well as residues M137 & T138 for benzocaine which can penetrate deeper into the pocket. It is also evident that van der Waals interactions are more significant than electrostatic forces (Fig. S3).

Since both drugs are uncharged and there are no aromatic residues available for pi-pi stacking or cation-pi interaction in the NavAb channel lumen, nonspecific binding based on van der Waals attraction to hydrophobic pockets seems to be the dominant force behind drug association in our simulations. Both the identified binding sites involve significant interactions with residues at positions previously shown to be important for block in eukaryotic channels [19], however this appears to be due to their location and hydrophobicity, rather than any specific chemical moieties that interact with benzocaine or phenytoin. Our results are consistent with previous studies that suggest that the local anesthetic site for tonic block is spread out over several residues and is hydrophobic in nature [32]. Despite the positions of binding being consistent with expectations from studies of eukaryotic channels, it is difficult to use the present results to directly rationalise the mechanisms of block in the eukaryotic case. There is a significant sequence difference between bacterial and eukaryotic channels in the central cavity, notably a lack of aromatic residues, as well as the bacterial channels being homotetramers. The lack of specific interactions found here contrasts with the large affinity differences obtained for phenytoin derivatives (containing different aromatic rings and substituents) in Nav1.7 [33], presenting a further warning that the mode of binding in bacterial channels may differ from that in the eukaryotic counterparts due to the different composition of the pore lining residues. Most experimental studies examine drug binding of the inactivated state, as this is in general stronger than that found for closed channels. The bacterial channels may represent a better model of tonic block to closed channels than inactivated channels as there is evidence this is not based upon aromatic association [32]. We hope that the interactions described here for NavAb can be experimentally tested via mutagenesis in the future.

### Drug binding affinities

To assess the strength of binding relative to bulk in each of the two sites identified in the unbiased and metadynamics simulations we employed the method of free energy perturbation. Using this, the binding free energy of each drug in each site was determined, from which the dissociation constant was calculated for comparison to experimental measurements. As shown in Table 1, phenytoin and benzocaine have comparable affinity for the NavAb activation gate. For both phenytoin and benzocaine binding in the activation gate was stronger than binding in the hydrophobic fenestration, as seen in the metadynamics simulations.

**Table 1:** Free energy of binding, and dissociation constants relative to bulk water for phenytoin and benzocaine at two sites in the NavAb central cavity.

		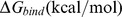	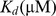
**Benzocaine**	Activation gate	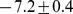	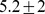
	Fenestration	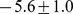	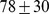
**Phenytoin**	Activation gate	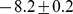	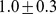
	Fenestration	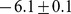	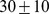

For benzocaine, published values for the dissociation constant in closed eukaryotic channels range from 0.3–1.2 mM [34]–[36] and a similar value (0.65 mM) is seen for NaChBac [9]. Benzocaine is known to bind within the central pore [19], and the current understanding of neutral drug-binding to the closed state supposes that binding is caused by hydrophobic interactions to a diffuse receptor spreading across several residues [32]. The dissociation constant relative to bulk water in our simulations is in the 5 – 100 μM range. While this does suggest stronger binding in the simulation, a direct comparison with experiment is complicated by a number of factors. Firstly, both benzocaine and phenytoin are seen to strongly partition into lipid bilayers [37] which can influence observed and simulation dissociation rates. Secondly, since it will not be present in open channels, the activation gate site will only be relevant if drug binding here can alter the open probability of the channel, something which is difficult to assess. Finally, binding of a single drug in the fenestration does not fully occlude the pore meaning that the binding of another drug to another subunit (likely at lower affinity) may be necessary to block the passage of 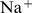. However, the similarity in the dissociation constants in the bacterial and eukaryotic Navs, and the consistency of the binding poses seen in our simulation with mutagenesis data, add support for NavAb representing a good model for tonic block of eukaryotic sodium channels by benzocaine. However, we note that since use-dependent block probably relies on an increase in affinity to the open/inactivated states [35], [36] facilitated by aromatic residues that are not conserved in NavAb [21], this channel is less likely to form a good model of use-dependent block.

Experimental dissociation constants for phenytoin binding to the closed channel are rare, due to its low solubility and affinity to this state. In eukaryotic channels, phenytoin has been shown to bind to inactivated channels with a dissociation constant in the range of 4 – 9 μM, with affinity up to 100-fold weaker in the closed state [31], [38], [39]. This provides similar agreement to our current result as for benzocaine. While there is some evidence for phenytoin binding on the external surface of the protein rather than in the pore [40] mutations to the internal local anesthetic binding site have shown these residues to be the major determinants of binding [20]. Our results suggest that if phenytoin does bind internally, NavAb may be a also be a reasonable model for closed-state binding of phenytoin.

### Drug entry through hydrophobic fenestrations

Drug passage through a hydrophobic pathway has long been hypothesized in sodium channels [8]. The bNav crystal structures all exhibit small fenestrations between homologous domains, which are potential routes for drugs to move into or out of closed or inactivated channels [13]–[17]. Previous molecular dynamics simulations in the absence of drugs have shown that the size if the fenestrations is likely to be sufficient to allow the passage of a small molecule such as benzocaine [24], [25]. Simulations with a drug present have the ability to directly assess the feasibility of drugs passing through these fenestrations and to examine the energetics and steps in the process. As noted above, benzocaine passes through a fenestration and binds on the outside surface of NavAb in one of our 125 ns equilibrium simulations, giving direct evidence that this molecule can move out of the channel via the fenestration. To determine the forces involved in such motion we used umbrella sampling to construct free energy profiles (potential of mean force, PMF) for the process.

**Figure 5:**
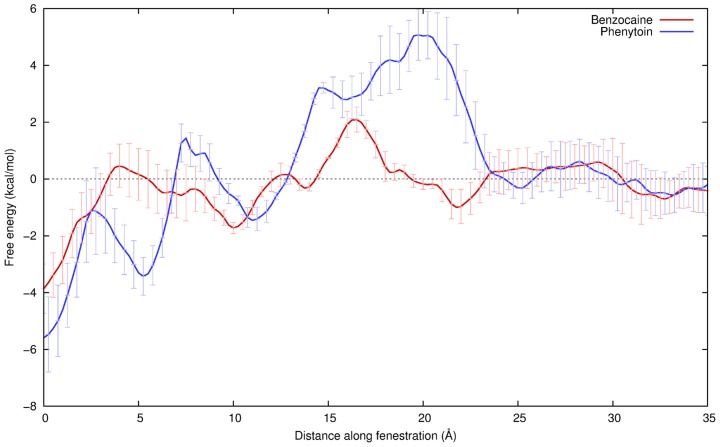
Potentials of mean force for benzocaine (red) and phenytoin (blue) moving from the pore axis (0 Å) to the lipid (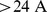) through one of the hydrophobic fenestration (10–20 Å). Snapshots of the drugs at positions along the fenestration are shown in Fig. 7.

As shown in Fig. 5, moving from the lipid bilayer into the central cavity is associated with a net negative change in free energy for both drugs. Both drugs show multiple minima inside the central cavity: at the activation gate (0 Å) and at the entrance to the fenestration (∼5,10 Å) in agreement with the equilibrium and metadynamics experiments. The site by the activation gate is about 2 kcal/mol more stable than that by the fenestration, reinforcing the results seen in our earlier calculations. Furthermore the barriers between the sites are in close agreement with what is seen in the metadynamics profiles. Comparatively, phenytoin shows a greater free energy change upon binding in the channel from the lipid than benzocaine, supporting the stronger binding affinity found here and in the literature. Previously we have shown that both drugs will partition into the bilayer from bulk water with a free energy change of −4.6 and −3.0 kcal/mol for benzocaine and phenytoin respectively [37]. Combining this with the free energy change for each drug to enter the channel from lipid seen in Fig. 5 yields a total binding free energy in close agreement (within 1 kcal/mol) with that found from the free energy perturbation calculation (table 1). While it may be feasible for phenytoin to enter through the fenestration, it faces larger barriers and will have a harder time leaving the pore via this route than does benzocaine.

**Figure 6:**
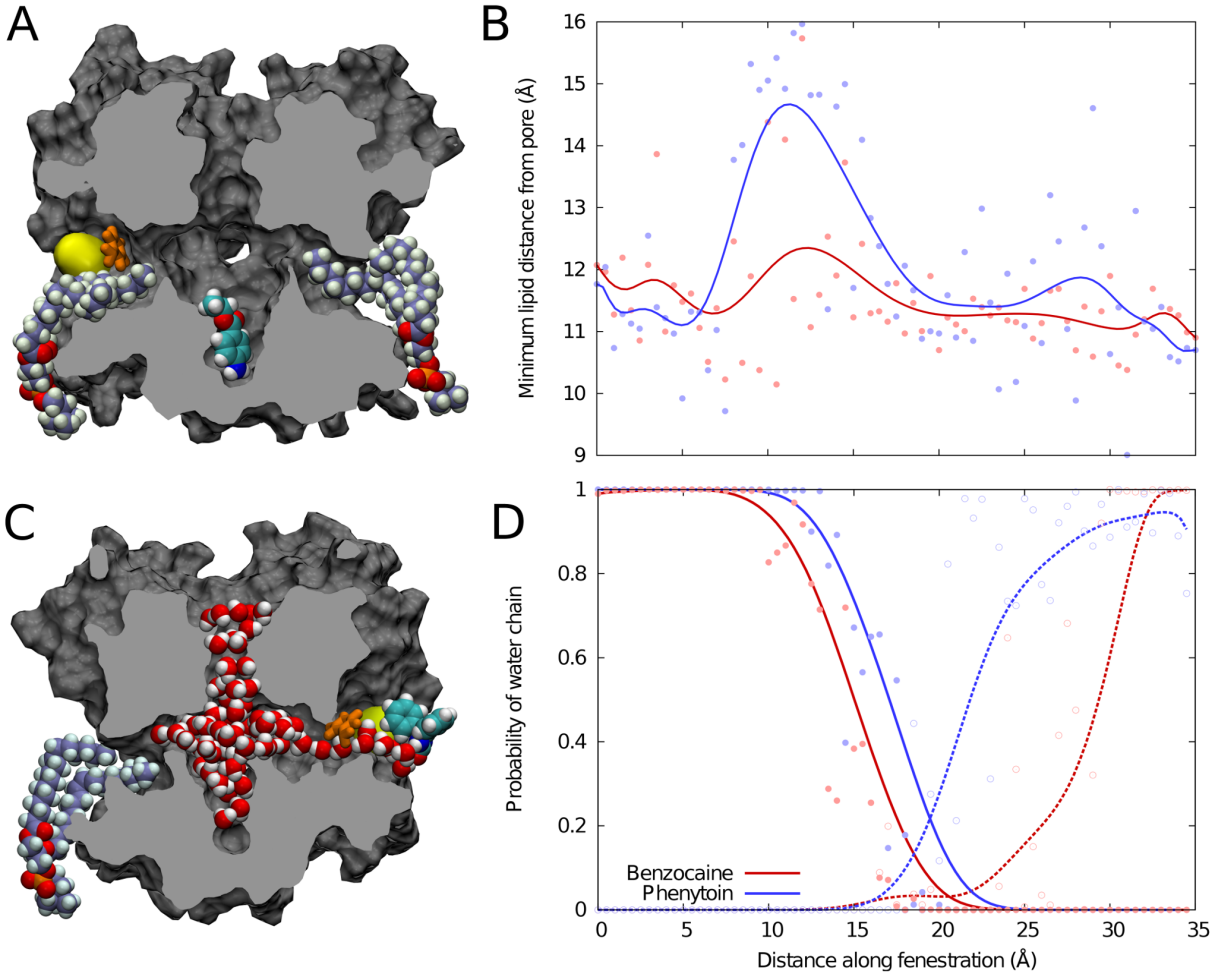
Lipid and water in the fenestrations as a function of drug position. (A) A representative snapshot showing lipid occupying the lateral fenestrations while benzocaine sits at its minimum energy position in the activation gate. The position of F203 (orange) and T138 (yellow surface) are also shown. (B) The extent to which lipid penetrates into the fenestration is plotted as a function of the position of each drug. Low values indicate extension further into the fenestration. (C) A snapshot showing an extreme example of a water chain extending from the channel lumen to phenytoin on the exterior surface of the protein. In most cases the water chain does not extend this far. (D) The probability that a continuous water chain extends from each drug back to the channel lumen as a function of drug position (solid lines). Also shown is the probability that a water chain extends from the drug directly to bulk water (dashed lines). In B and D the data for individual windows are shown in points and a moving average of 5 data points is indicated by the line.

Movement of benzocaine and phenytoin through the fenestration is associated with extension of water chains from the channel lumen into the fenestration and possible retraction of resident lipid tails. Without the drug present, lipids extend into each fenestration for the majority of our simulations (eg Fig. 6A) as suspected from the crystal structure [13]. Benzocaine can move past the resident lipid tails without significantly displacing them as shown in Fig. 6B. However, the bulkier phenytoin cannot fit in the mid part of the fenestration with the lipid tails, and these have to move out of the way as the drug passes, similar to what is seen for isoflurane [26]. Examples in which the drug and lipid co-exist in the fenestration are shown in Fig. S4. Passage of the drugs through the inner part of the fenestration is also stabilised by a single-file water chain that extends from the channel lumen to contact polar moieties on the drug. This water chain tends to form up to the point that the drug passes the bulky F203 residue as seen in Fig. 6D, but remarkably, at times this chain extends the entire length of the fenestration when the drug resides on the outside surface of the protein (Fig. 6C). As seen in Fig. 6D the water chain tends to extend further to the more polar phenytoin than it does to benzocaine, similar to what is seen in simulations of the partitioning of these drugs into lipid bilayers [37].

**Figure 7:**
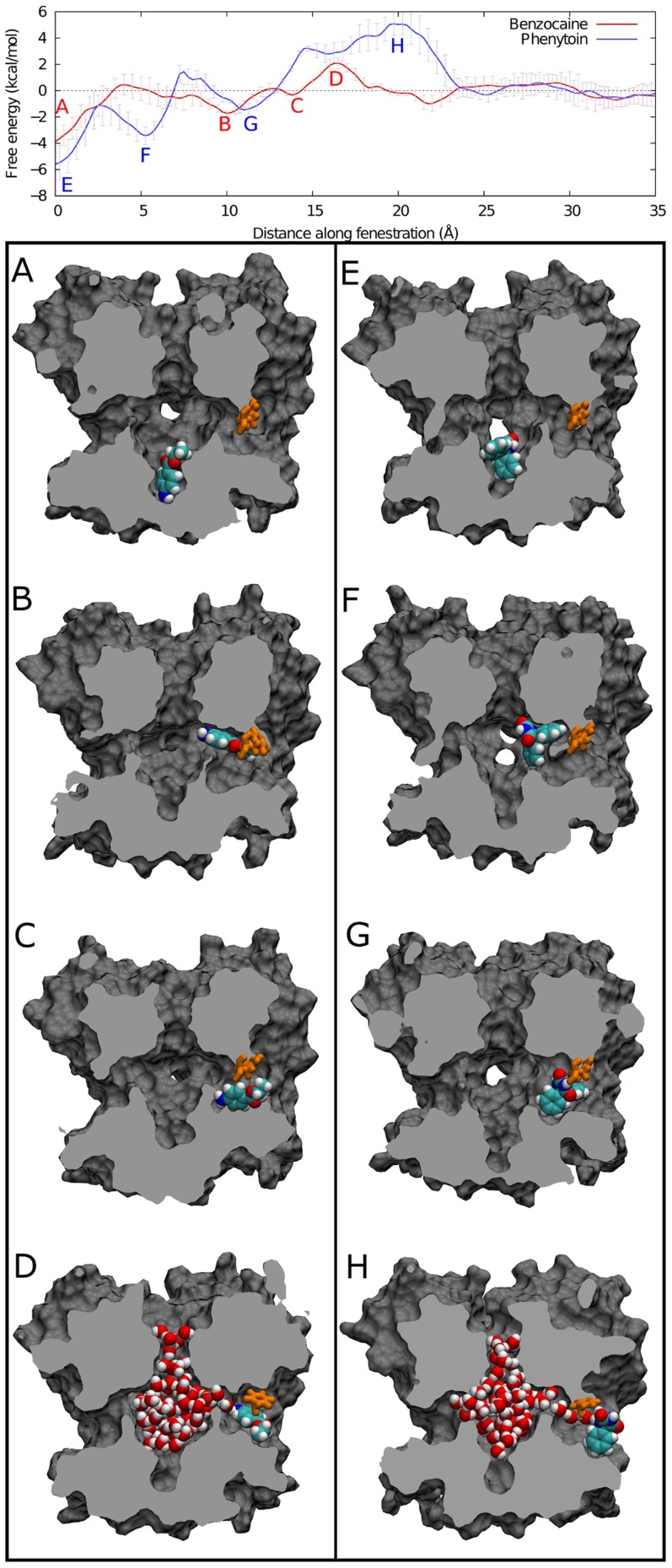
Snapshots from the umbrella simulations are shown that represent important points in the PMF for benzocaine (A–D) and phenytoin (E–H). The global minimum for each drug is at the activation gate (A,E), and the drug positions here replicate the binding poses seen in the equilibrium simulations. The same is true for the second minimum (B, F), which shows binding in the fenestration. A further hydrophobic pocket supports the drugs in the outer fenestration (C,G). At the external entrance to the fenestrations, the drugs have to pass the bulky phenylalanine residue (orange) and are at their most dehydrated creating the largest barrier in the pathway (D,H). Example snapshots show that even at this point water chains extending to the channel lumen are sometimes present.

Fig. 7 shows representative snapshots of the system at points corresponding to minima on the PMF. Two minima identified in the equilibrium simulations and metadynamics are reproduced here - namely in the activation gate (0 Å) and in the fenestration (11 Å). The bulky phenylalanine that constricts the fenestration (F203) appears to hinder the passage of both drugs as seen by the barriers in the region 15 Å–18 Å, but also contributes to pi-pi stacking with the aromatic moieties. Once on the outside surface of the protein, both drugs exhibit enduring association with F203 (pi-pi stacking) as well as with T138 (yellow in Fig. 6), the one polar residue near the mouth of the fenestration to which it can hydrogen bond.

Moving from the bulk lipid into the fenestration requires the drugs to move into the hydrophobic core of the membrane, something previously shown to be unfavourable [37]. Benzocaine is seen to slide slowly down the side of the protein from its preferred position near the lipid headgroups toward the mouth of the fenestrations. Because of this it only makes significant contact with water molecules from bulk when far from the protein (Fig. 6D dashed line). The interaction of benzocaine with the protein helps to reduce the energetic barrier for penetrating deep into the bilayer. In contrast, phenytoin has greater difficulty in penetrating into the centre of the bilayer as seen by the bump in the free energy profiles at around 21 Å. This corresponds to a sudden move from the lipid headgroups to the mouth of the fenestration and the rapid breakage of the water chain to the bulk water (Fig. 6D dashed line). Dehydration of the drug as it enters the bilayer is the principle cause of this barrier. While the hieght of the barrier is significant (∼5 kcal/mol), it is not as large as in the absence of protein [37].

Accurately converging the right hand side of the free energy profiles is difficult. Once the drug leaves the fenestration it can sample more positions at the given coordinate value and there are a number of slow motions that are hard to sample in the 30 ns of simulation we allow for each drug position. These slow motions include lipids entering/leaving the fenestrations, the drug moving between leaflets of the bilayer and the formation and breakage of water chains. Although the PMF does not change significantly when we extend our simulations (Fig. S5) we have noted a dependence upon the starting coordinates used in each window. Given the difficulty in sampling this region and the long timescale of some of the important motions, some care should be taken in reading quantitative values from the free energy profiles. However, the close agreement of our PMF to the metadynamics and FEP experiments, particularly in the interior of the channel and fenestration, provides confidence that this area is well sampled.

## Discussion

Using molecular dynamics simulations we have characterised the likely binding sites for benzocaine and phenytoin inside the central cavity of NavAb, with 3 independent simulation methods yielding consistent results. Both drugs are found to bind either in the hydrophobic pocket formed by the activation gate or at the entrance to the hydrophobic fenestrations. In both cases, the drugs interact with residues found to be important in binding in eNavs. But, we find this association is due to non-specific hydrophobic interactions meaning that binding occurs due to the overall architecture of the central cavity rather than interactions to specific residues. Although our simulations clearly identify the likely drug binding sites in the channel, it is not yet clear if the binding of more than one drug is necessary to occlude the pore or prevent channel opening. Our simulations also provide a plausible rationalisation for the differences seen between tonic and use dependent block, as interaction of cationic drugs with aromatic residues are essential in the use dependent case but not for resting state block [21], [32]. It is possible that the most likely binding position in the resting channel could be at the activation gate as found here, but this will be abolished upon channel activation allowing the drug to move close to the aromatic residues present in eNavs. If this hypothesis is correct, mutation of residues involved only in binding at the activation gate would be expected to change the affinity of tonic block, but not use-dependent block. Unfortunately, the lack of aromatic residues in the pore and the slow channel kinetics seen for NaChBac make these channels less ideal for modelling use-dependent block in either simulation or experiment - but they still may allow for modeling of eukaryotic tonic block. We note that the dissociation constant for tonic block by benzocaine in eukaryotic channels [41] closely matches that for bacterial channels [9].

Our results also show that the hydrophobic fenestrations seen in NavAb can serve as conduits for benzocaine and phenytoin to enter the central cavity and thus can form the long hypothesised hydrophobic entrance route [8]. There are three lines of evidence supporting this conclusion: (i) benzocaine exits through a fenestration during one of the equilibrium simulations, (ii) the energy barriers to reach the outside of the protein are small as found from metadynamics simulations, and (iii) the PMF plots indicate small barriers for drug entry, although larger barriers are present for penytoin than for benzocaine. The main barriers to passage through the fenestration appear to be physical constrictions in the fenestrations which is consistent with experiments in eukaryotic channels, in which analogs of benzocaine show slower rates of dissociation from sodium channels when they have bulkier or longer moieties attached [42]. It has been suggested that there may be scope to exploit any differences in the size of the fenestrations in different eNav subtypes or in different functional states to rationally design sub-type or state dependent channel blocker [16], [14], however, our previous simulation have suggested there is little state dependent difference in fenestration size [24]. In addition the concept of drug entry through the fenestration may be further tested using mutations in this area. Surprisingly, the potential entropic issues associated with the drug finding the small fenestration entrance appear to be small. The fact that lipids are seen to exchange in long simulations, that they are replaced by drug and water, and that polar residues on the surface of the protein can attract drugs from the bilayer may all help to overcome this potential barrier. Polar residue in this region (T138,Y142) are well conserved in bNavs and it would be interesting to see if their mutation altered the kinetics of drug entry.

The large degree of sequence similarity in the pore forming regions of eNavs makes the design of subtype selective channel blockers a difficult goal. Elucidating the mode of entry and binding of existing channel blockers is an important step toward achieving this aim. Here we have been able to do this for benzocaine and phenytoin in a bacterial channel, but it is not clear how much about binding in eukaryotic channels can be inferred from this given the significant differences between these families of proteins. For example, the nature of binding seen here is likely to be less specific than that in the eukaryotic homologues. Additional functional studies of drug block in bacterial channels, increased availability of structural information about the different functional states of bacterial channels, reliable models of eukaryotic channels and detailed investigations of specific drug-residue interactions in tonic and use-dependent block will all aid progress toward the design of subtype selective channel blockers.

## Methods

### Simulation systems

Two simulation systems were set up based on the NavAb sodium channel with coordinates obtained from the protein database: a closed/pre-open channel; PDB accession code 3RVY [13] and a potentially inactivated channel; PDB accession code 4EKW [14]. The voltage sensors (residue numbers below 115) were removed from both systems to reduce the system size and computational load, as experimental studies have shown this still yields a functional channel in other bNavs [43], [44]. Each system was placed in a pre-equilibrated POPC lipid bilayer, and solvated in a 72×72×82 Å box of TIP3P water with 250 mM NaCl. A picture of one such system is given in Fig. S6. The systems were equilibrated as for our previous simulations [45]: the protein was first held fixed while water and lipid were allowed to equilibrate for 2 ns. Then the protein alpha carbons were then restrained by a harmonic potential with force constant reducing from 10 kcal/mol to 0.1 kcal/mol in 4 steps over 10 ns. To get lipid to rapidly take up position in the fenestrations as indicated in the crystal structure, four lipid tails were restrained in the fenestration during the equilibration period, but this restraint was removed in subsequent simulations and lipids remained present in the fenestrations for the duration of the equilibrium simulations. All simulations were run with periodic boundary conditions with constant pressure of 1 atm and temperature 298 K maintained using a Langevin Piston and Langevin dynamics respectively. The CHARMM 27 force field was used for proteins [46] and CHARMM36 lipid [47] and ion parameters were taken from Joung and Cheatham [48]. A 2 fs timestep was used with all bonds to hydrogen atoms fixed. Parameters for benzocaine and phenytoin were chosen from those best replicating water/octanol partition data as described in our previous work [37].

### Equilibrium simulations

In order to gain a first idea of the likely position of drug binding in the central pore cavity, three simulations lasting 125 ns each were run for each protein/drug system (yielding a total of 12 simulations). In each of these the drug was placed in the center of the cavity with a different random orientation. Once complete, the first 25 ns was excluded from further analysis to allow for equilibration of the drug in the channel, and the three trajectories for each protein/drug system were combined. Cluster analysis was performed on the drug position in the combined trajectory (after aligning the protein coordinates) using the quality threshold algorithm to locate the most commonly occupied drug positions. For this purpose we clustered according to RMSD of the drug coordinates using a cutoff of 3 Å. To determine which residues the drug interacts with in potential binding sites, the NAMDEnergy plugin was used to measure the interaction between the drug and chosen residues for selected subsets of the trajectory corresponding to each cluster.

### Metadynamics

To better assess if the equilibrium simulations had sampled the most likely drug binding poses, metadynamics [49], [50] was utilised to force the drugs to sample the entire pore. For this purpose, a metadynamics bias was applied to three collective variables, which described the x, y, and z coordinates of the drug centre of mass inside the central cavity. A restraint was applied to the backbone atoms of the NavAb residues on the S5 helices, with a spring constant of 0.2 kcal/mol. This allowed Cartesian coordinates to be used for the metadynamics collective variables. Well tempered metadynamics [51] with a bias-factor of 10 was applied using the PLUMED package [52]. To improve sampling, boundaries were imposed using flat bottomed harmonic potentials to prevent the drug from passing out of the fenestrations or beyond the activation gate. Boundaries were also employed to focus sampling on a single quarter of the x,y plane making use of the inherent four fold symmetry of the protein. Gaussians with a height and width of 0.25 kcal/mol were deposited every 1000 timesteps, equivalent to 2 ps. Results represent 400 ns of simulation for each drug.

### Free energy perturbation

Free energy perturbation [53] was used to calculate the free energy difference between a solvated and a bound drug. The perturbation experiment was performed in the forward (solvated to bound) and reverse (bound to solvated) directions to assess reproducibility of the calculations. In addition two independent sets of simulations were made, one in which the solvated drug appeared in the bulk water region of the same protein simulation, and one in which the solvation energy of the drug was determined in a completely separate simulation of a water and ion containing box, and the quoted results represent the average of all the simulations. These calculations used the native alchemical free energy module present in the NAMD software in only closed the NavAb. The transformation was stratified into 40 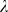 windows, differing by 0.025. For each window, 0.5 ns of equilibration was performed, before ensemble averaging was turned on for 2 ns. A softcore potential [54] was used to avoid explosively large energy values at each end of the 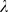 scale when one of the drugs was nearly annihilated. This scaled down the electrostatic interactions from 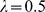 to 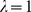, and the van der Waals interactions from 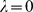 to 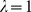 for annihilated particles. During free energy perturbation, the free energy wells that define binding sites are flattened as a drug disappears and particle interactions are scaled down, so positional restraints were applied to bound drugs to ensure they remain in the relevant region of the protein. A flat bottomed 2 Å radius harmonic potential with 5 kcal/mol spring constant was applied to keep the drug centre of mass close to the centre of mass of a set of residues determined in the equilibrium simulations. Each restraint enclosed the drug in a sphere of radius 2 Å using a harmonic potential with a 5 kcal/mol spring constant. To achieve complete sampling in bulk an identical restraint held the solvated drugs to a dummy atom in water, and the energy values were corrected for the associated loss in translational entropy.

The equilibrium constant of the drug binding to each site were calculated using the following relation [55]:
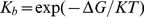
(1)where 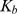 is the binding affinity, 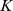 is the Boltzmann constant, 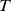 is the absolute temperature and 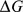 is the free energy change for the drug to enter the binding site from bulk water as found in the free energy perturbation simulation. The dissociation constant 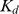 was calculated using the relation 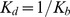.

### Umbrella sampling

The energy profile for drugs to pass through the lateral fenestrations from the channel cavity into the lipid bilayer was determined using umbrella sampling [56]. Starting coordinates for these simulations were generated in two ways. In the first, steered molecular dynamics was used to pull the drug centre of mass through the fenestration and into the lipid at a rate of 2 Å/ns, from which coordinates were saved at 2 Å intervals. In the second, the drug was equilibrated in bulk lipid and starting coordinates were generated by pulling the drug back into the fenestration. In both cases the drug position was defined from the distance from a plane passing through the pore axis perpendicular to the direction of the fenestration (equivalent to the x-coordinate of the drug center of mass in our simulation system). The position of the centre of mass of the drug was then restrained in 0.5 Å intervals along the fenestration using PLUMED [52] to create a harmonic potential with force constant 3 kcal/mol. Additional restraining walls were put in place using a harmonic external potential with a spring constant of 10 kcal/mol to focus sampling on a 20 Å-wide portion of the lipid bilayer (defined by the y-coordinate of the drug center of mass). Each window was run for 30 ns of simulation, with the first 5 ns used as equilibration. Simulations using the first set of stating coordinates were used for the left hand part of the profile (x ≤ 16 Å), while those with the second set of starting coordinates for the right hand part of the profile (x ≥ 12 Å), with both data sets used for the overlapping region. This yielded a total of 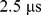 of simulation per drug. Three additional windows with a force constant of 10 kcal/mol, were required for phenytoin to ensure good overlap between the windows midway through the hydrophobic fenestration. Collective analysis of the data was made using the Weighted Histogram Analysis Method [57], [58], using the implementation of Grossfield [59], to produce a one dimensional potential of mean force. Uncertainties were estimated from the standard error in the mean found when dividing the data into 3 blocks and convergence of the profiles is discussed in the supplementary material.

## Supporting Information

Figure S1 **Orientations of the drugs as a function of time during the metadynamics simulations.**
(PDF)

Figure S2 **Representative snapshots of the binding poses of benzocaine phenytoin.**
(PDF)

Figure S3 **Drug-protein interaction energy decomposition.** The interaction energies for benzocaine and phenytoin and residues in NavAb are decomposed into van der Waals and electrostatic components.
(PDF)

Figure S4 **Snapshots from umbrella simulations.** These show benzocaine and phenytoin occupying a hydrophobic fenestration at the same time as a lipid molecule from the bilayer.
(PDF)

Figure S5 **Convergence of the potentials of mean force (PMF).**
(PDF)

Figure S6 **Cross-section of the simulation system used in this study.**
(PDF)
