# Supplementary material for: Locating the Route of Entry and Binding Sites of Benzocaine and Phenytoin in a Bacterial Voltage Gated Sodium Channel
Source: PLoS Comput Biol. 2014 Jul 3;10(7):e1003688. doi: 10.1371/journal.pcbi.1003688 (PMC4084639; doi:10.1371/journal.pcbi.1003688)
Supplement: Figure S1 — Orientations of the drugs as a function of time during the metadynamics simulations. (PDF) [file pcbi.1003688.s001.pdf]

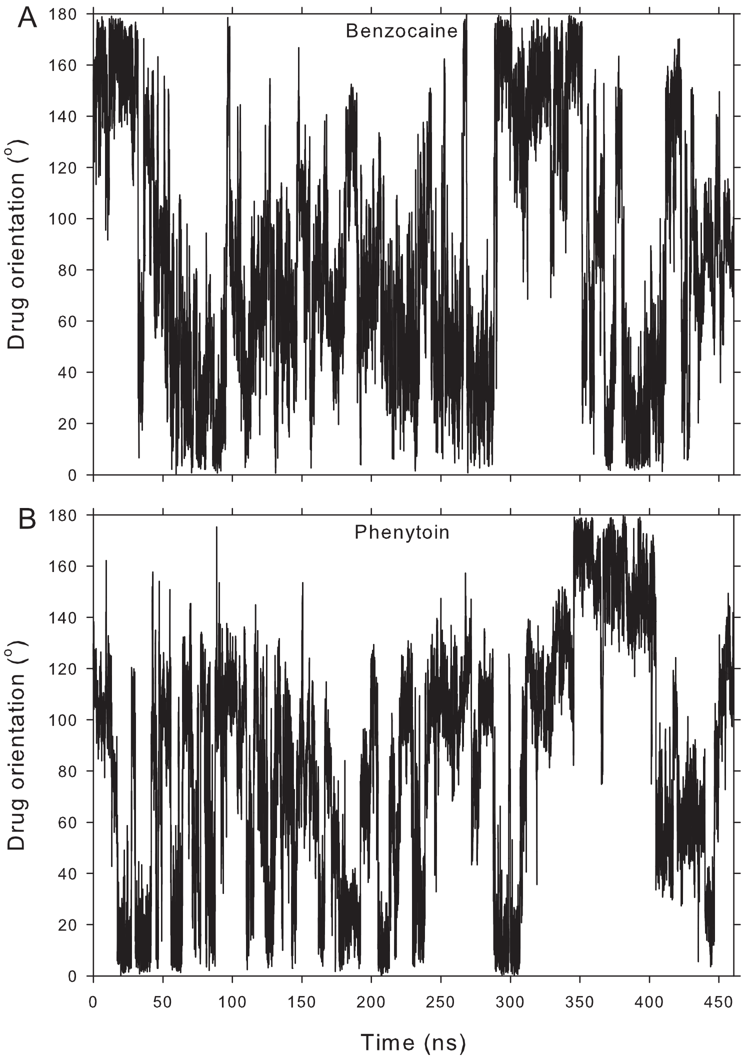

Figure S1: Drug orientation versus time for (A) benzocaine and (B) phenytoin. The angle between the channel axis and a line joining 2 of the atoms in the drug is plotted. For benzcaine this line is defined from the amide nitrogen to the ester carbon . For phenytoin we use the central carbon joining the three aromatic rings and the most distant carbon on one of the phenyl rings.
