# Supplementary material for: Locating the Route of Entry and Binding Sites of Benzocaine and Phenytoin in a Bacterial Voltage Gated Sodium Channel
Source: PLoS Comput Biol. 2014 Jul 3;10(7):e1003688. doi: 10.1371/journal.pcbi.1003688 (PMC4084639; doi:10.1371/journal.pcbi.1003688)
Supplement: Figure S2 — Representative snapshots of the binding poses of benzocaine phenytoin. (PDF) [file pcbi.1003688.s002.pdf]

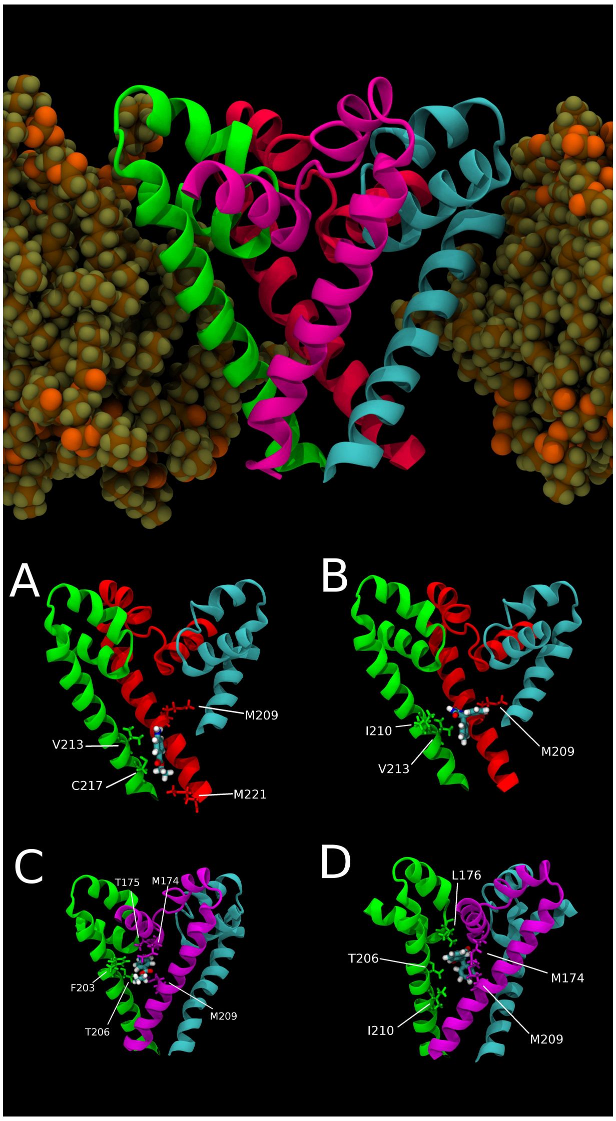

Figure S2: Representative snapshots of the binding poses of benzocaine (A,C) and phenytoin (B,D) in the activation gate (A,B) and the fenestration (C,D). The top panel shows the NavAb system (coloured helices) in a lipid bilayer for reference. Residues that contribute the most energy to binding in each of these sites, also shown in Figure 2, are highlighted. In each case, binding arises from hydrophobic association between the drug and a pocket of the NavAb central cavity.
