# Supplementary material for: Locating the Route of Entry and Binding Sites of Benzocaine and Phenytoin in a Bacterial Voltage Gated Sodium Channel
Source: PLoS Comput Biol. 2014 Jul 3;10(7):e1003688. doi: 10.1371/journal.pcbi.1003688 (PMC4084639; doi:10.1371/journal.pcbi.1003688)
Supplement: Figure S3 — Drug-protein interaction energy decomposition. The interaction energies for benzocaine and phenytoin and residues in NavAb are decomposed into van der Waals and electrostatic components. (PDF) [file pcbi.1003688.s003.pdf]

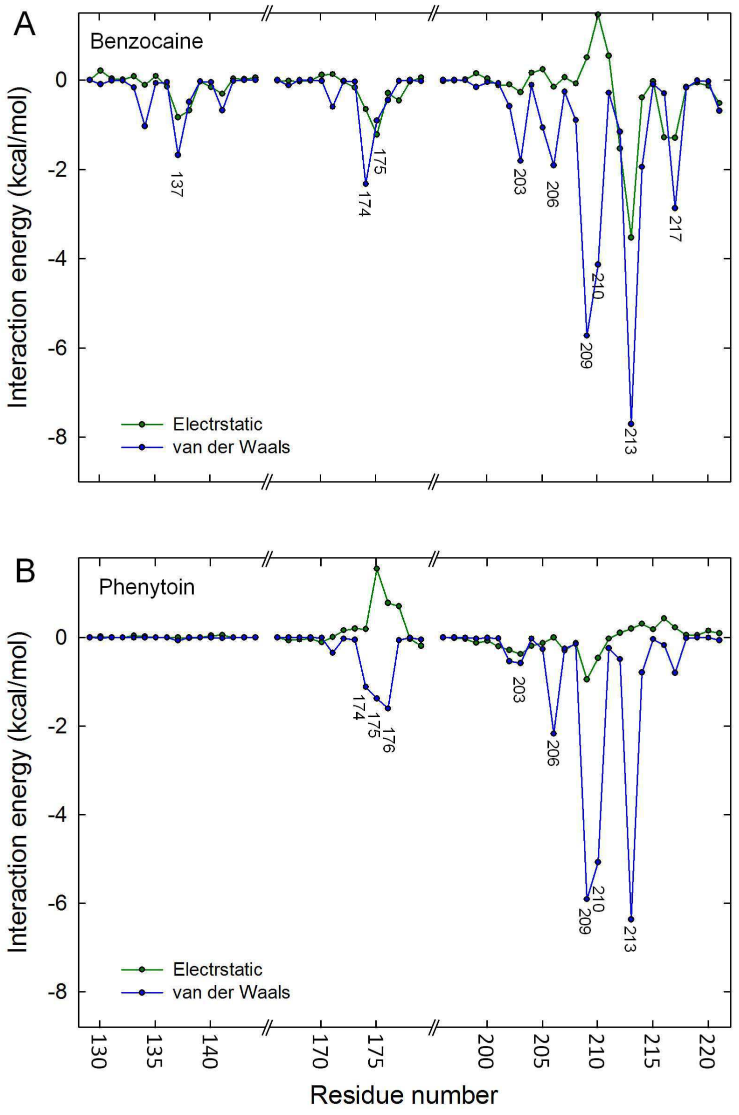

Figure S3: Drug-protein interaction energy decomposition. The interaction energies for (A) benzocaine and (B) phenytoin and residues in NavAb are decomposed into van der Waals (blue) and electrostatic (green) components. Values are calculated by for trajectory frames corresponding to when the drug is in one of the identified clusters, meaning the results average over the individual binding positions. Due to the four-fold symmetry of NavAb, values are averaged across all four subunits. These energies show that van der Waals forces are greater than electrostatic forces in a majority of the interactions contributing to binding.
