# Supplementary material for: Locating the Route of Entry and Binding Sites of Benzocaine and Phenytoin in a Bacterial Voltage Gated Sodium Channel
Source: PLoS Comput Biol. 2014 Jul 3;10(7):e1003688. doi: 10.1371/journal.pcbi.1003688 (PMC4084639; doi:10.1371/journal.pcbi.1003688)
Supplement: Figure S4 — Snapshots from umbrella simulations. These show benzocaine and phenytoin occupying a hydrophobic fenestration at the same time as a lipid molecule from the bilayer. (PDF) [file pcbi.1003688.s004.pdf]

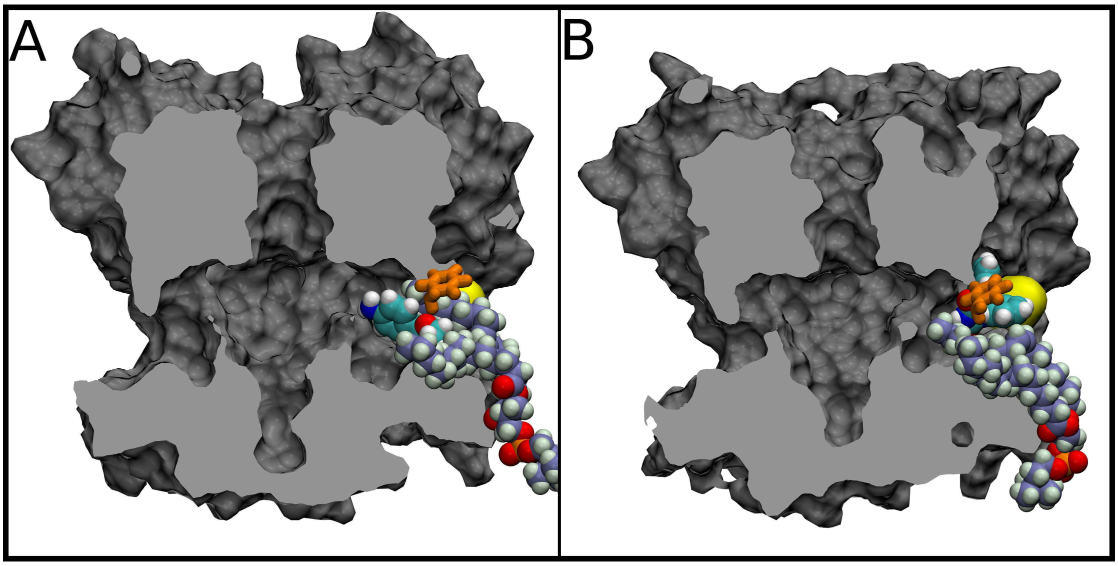

Figure S4: Snapshots from umbrella simulations showing benzocaine (A) and phenytoin (B) occupying a hydrophobic fenestration at the same time as a lipid molecule from the bilayer. The drugs are oriented such that their polar moieties point into the NavAb central cavity so that they can contact an aqueous environment. The lipid tails from the cytosolic leaflet could access the hydrophobic fenestration while the drug was present, and were seen to move in and out of the fenestration around the drug.
