# Supplementary material for: Locating the Route of Entry and Binding Sites of Benzocaine and Phenytoin in a Bacterial Voltage Gated Sodium Channel
Source: PLoS Comput Biol. 2014 Jul 3;10(7):e1003688. doi: 10.1371/journal.pcbi.1003688 (PMC4084639; doi:10.1371/journal.pcbi.1003688)
Supplement: Figure S5 — Convergence of the potentials of mean force (PMF). (PDF) [file pcbi.1003688.s005.pdf]

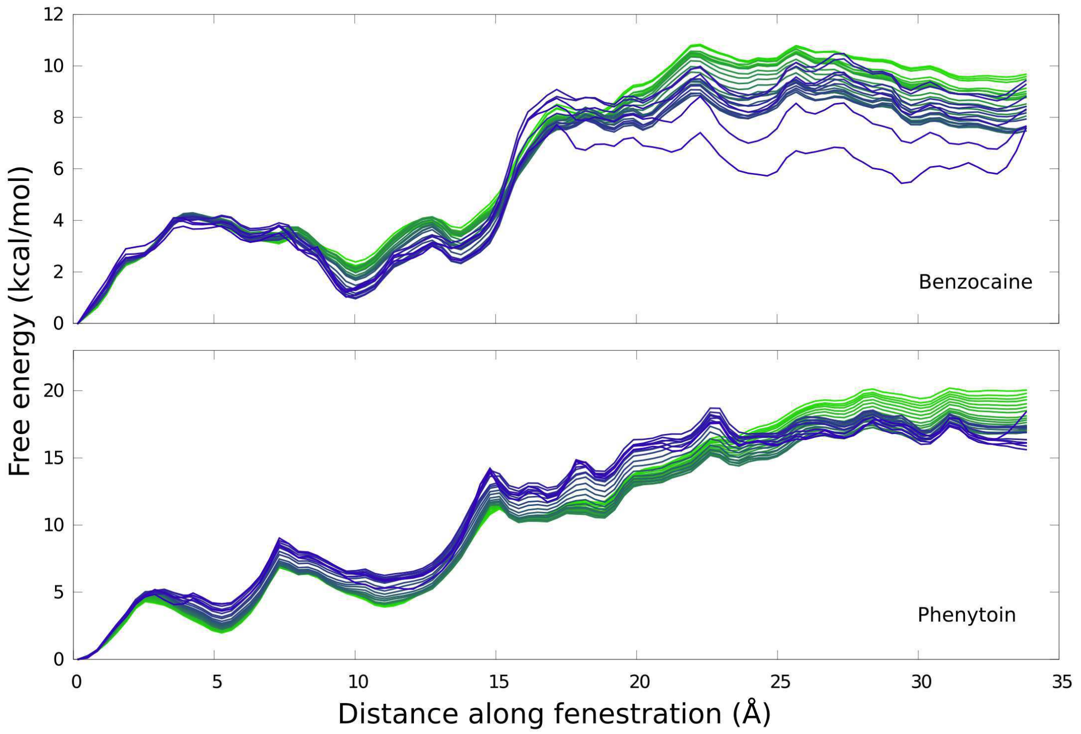

Figure S5: Convergence of the potentials of mean force (PMF). The PMF for benzocaine (top panel) and phenytoin (bottom panel) moving through a hydrophobic fenestration, generated from successive data sets from umbrella sampling. The final nanosecond of data is shown in blue, and each line previous (changing to green) includes the prior nanosecond. These potentials show that after ~5 ns the potentials are not rapidly changing, and this was the basis for discarding the first 5 ns of data as equilibration. Inclusion of more than 25 ns of data does not significantly change the PMF, indicating either that ther profile is well converged or that it is only influenced by slow motions that are difficult to capture in the timescale of these simulations.
