# Supplementary material for: Locating the Route of Entry and Binding Sites of Benzocaine and Phenytoin in a Bacterial Voltage Gated Sodium Channel
Source: PLoS Comput Biol. 2014 Jul 3;10(7):e1003688. doi: 10.1371/journal.pcbi.1003688 (PMC4084639; doi:10.1371/journal.pcbi.1003688)
Supplement: Figure S6 — Cross-section of the simulation system used in this study. (PDF) [file pcbi.1003688.s006.pdf]

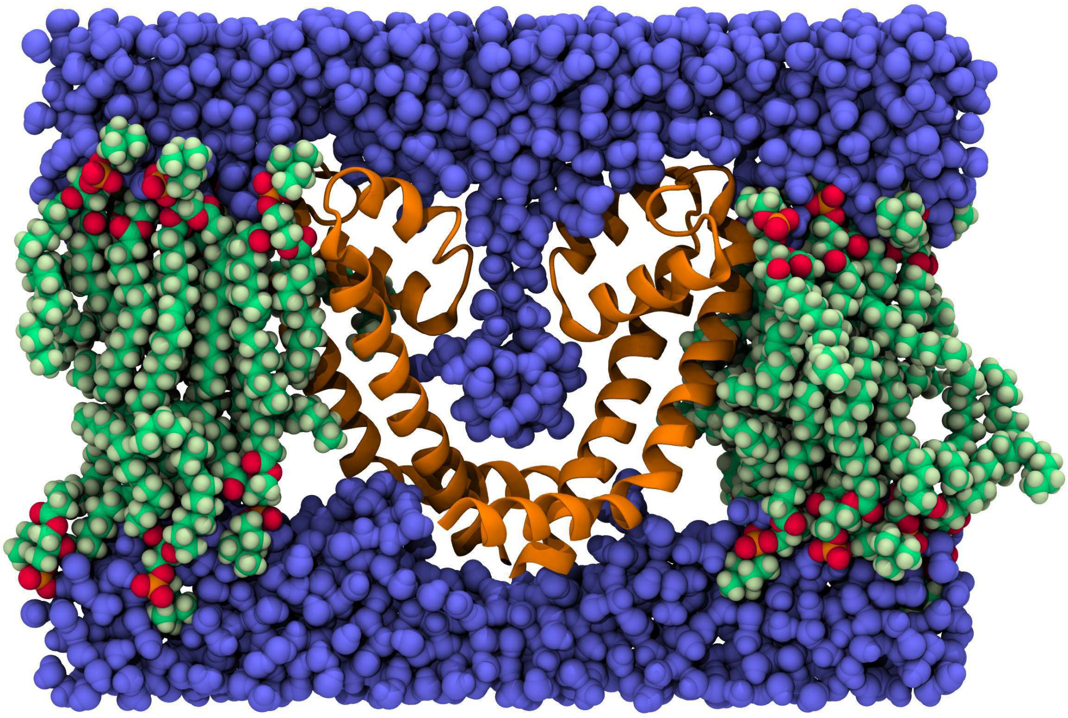

Figure S6: Cross-section of the simulation system used in this study. To of the four protein subunits of the NavAb protein is shown in orange helices, with a lipid bilayer (green and red) and water (blue), which extends into the protein's aqueous central cavity. Salts (NaCl), which are present at physiological concentrations, are omitted for clarity.
